# Nest Predation Deviates from Nest Predator Abundance in an Ecologically Trapped Bird

**DOI:** 10.1371/journal.pone.0144098

**Published:** 2015-12-01

**Authors:** Franck A. Hollander, Hans Van Dyck, Gilles San Martin, Nicolas Titeux

**Affiliations:** 1 Behavioural Ecology & Conservation Group, Biodiversity Research Centre, Earth and Life Institute (ELI), Université catholique de Louvain (UCL), Louvain-la-Neuve, Belgium; 2 Unité Protection des plantes et écotoxicologie, Département des Sciences du Vivant, Centre wallon de Recherche agronomiques, Gembloux, Belgium; 3 Environmental Research and Innovation (ERIN), Luxembourg Institute of Science and Technology (LIST), Esch-sur-Alzette, Luxembourg; 4 InForest Joint Research Unit (CSIC-CTFC-CREAF), Forest Sciences Centre of Catalonia (CEMFOR-CTFC), Solsona, Spain; University of Sydney, AUSTRALIA

## Abstract

In human-modified environments, ecological traps may result from a preference for low-quality habitat where survival or reproductive success is lower than in high-quality habitat. It has often been shown that low reproductive success for birds in preferred habitat types was due to higher nest predator abundance. However, between-habitat differences in nest predation may only weakly correlate with differences in nest predator abundance. An ecological trap is at work in a farmland bird (*Lanius collurio*) that recently expanded its breeding habitat into open areas in plantation forests. This passerine bird shows a strong preference for forest habitat, but it has a higher nest success in farmland. We tested whether higher abundance of nest predators in the preferred habitat or, alternatively, a decoupling of nest predator abundance and nest predation explained this observed pattern of maladaptive habitat selection. More than 90% of brood failures were attributed to nest predation. Nest predator abundance was more than 50% higher in farmland, but nest predation was 17% higher in forest. Differences between nest predation on actual shrike nests and on artificial nests suggested that parent shrikes may facilitate nest disclosure for predators in forest more than they do in farmland. The level of caution by parent shrikes when visiting their nest during a simulated nest predator intrusion was the same in the two habitats, but nest concealment was considerably lower in forest, which contributes to explaining the higher nest predation in this habitat. We conclude that a decoupling of nest predator abundance and nest predation may create ecological traps in human-modified environments.

## Introduction

Organisms have evolved to use environmental cues, such as vegetation properties or abundance of predators, as a proximate indicator of habitat quality during habitat selection [1]. In human-modified environments, these proximate indicators may deviate from the ultimate factors affecting survival and reproduction [2,3]. Organisms that rely on such cues may then be trapped and preferentially use habitats where survival or reproductive outcomes are lower than in other available habitats [4,5]. Several studies on ecological traps focused on birds and frequently report lower nest success due to higher impact of nest predators in the preferred habitat types [6–8]. To explain this pattern, it has often been suggested that organisms fail to avoid areas with the highest densities of nest predators [9,10]. However, most studies on ecological traps only focus on the numerical response of nest predators (i.e. nest predator abundance) and few of them explicitly addressed the functional response of nest predators that results from the complex interaction between habitat structure and the behaviour of both the nest predators and the prey [11–14]. This may reduce the ability to understand the genuine predation-related mechanisms underlying ecological traps in human-modified environments.

An ecological trap was recently shown for the Red-backed shrike (*Lanius collurio*) [15]. In Belgium as in other parts of Europe, this species uses two different habitat types in mixed farmland-forest landscapes [15–17]. Shrikes used to breed in open areas under a management regime of extensive farming, but modern forest management has created open areas in spruce plantation forests that offer a novel habitat for the species. Based on intensive field sampling and experiments, our previous studies have shown that this species prefers breeding in open areas in spruce plantation forests over adjacent farmland habitat even though nest success (i.e. the production of fledging offspring) and reproductive performance (i.e. offspring quality and quantity) are higher in farmland [15,18]. Food limitation for the shrikes in forest provides a functional explanation for the lower offspring quality and quantity [19,20]. However, the proximate explanation for attraction to the forest habitat has not yet been resolved and the mechanisms that induce a lower nest success through a higher proportion of brood failures in this preferred habitat remain unknown.

At this stage, there are two alternative predation-related hypotheses to improve our mechanistic understanding of this ecological trap: a ‘numerical response’ hypothesis and a ‘functional response’ hypothesis. Corvids are the main nest predators of the shrike, while other birds or small mammals are only occasional predators [21–23]. Roos and Pärt [24] showed that the spatial distribution of the Red-backed shrike in the landscape is related to the distribution of corvids and they concluded that shrikes are able to use these nest predators as an indicator of habitat quality and to preferentially settle in habitats with fewer corvids. For that reason, it is unlikely that shrikes fail to avoid areas with the highest densities of nest predators in our study system. However, the distribution and relative abundance of corvids may change over the course of the season [22]. Hence, a first hypothesis is that shrikes are able to settle in forest habitats with fewer nest predators after their spring migration, but that a seasonal change in corvid abundance induces higher nest predator abundance in the preferred forest habitat than in farmland when shrike nests are depredated by corvids. This seasonal change in nest predator abundance may then provide an explanation for the attraction to the forest habitat and a higher proportion of predation-attributed brood failures in that habitat (i.e. the ‘numerical response’ hypothesis).

Human-induced changes in habitat structure may also favour nest predator activity and increase their impacts without actual changes in nest predator abundance [25,26]. Hence, a habitat with few nest predators may not necessarily be associated with higher nest success than an alternative habitat where nest predators are more abundant [27–30]. For instance, vegetation composition and structure in farmland and forest habitat types may mediate the ability of the shrikes to conceal their nests or to behave cryptically during food provisioning to the nest, which may, in turn, facilitate nest disclosure for visually-oriented nest predators like corvids [31]. Food limitation may also interact with nest predation [11,32]. In our study system, food limitation affected the rate at which parents delivered prey items to the nestlings and induced lower offspring quality in forest than in farmland [19]. Nest disclosure for predators may then be promoted by the hunger state of the nestlings through increased begging activities [33]. As parent shrikes dedicate more time to foraging activities in forest [19], they could be less able to trade-off their time budget for food provisioning against nest defence [32,34]. If nest defence varies with the perceived quality of the offspring as in several passerine species [35], food limitation may also indirectly increase predation on shrike nests in forest. A second hypothesis of a decoupling of nest predator abundance and their functional response is consistent with both attracting shrikes to the forest habitat with fewer nest predators and reducing their nest success in that habitat (i.e. the ‘functional response’ hypothesis).

To test the two alternative hypotheses, we first sampled corvid abundance in both habitat types during the settlement period and the breeding period of the shrikes to evaluate whether nest predator abundance differs between habitats and whether potential differences change over the course of the season. Second, we quantified nest predation using artificial and actual shrike nests in both habitat types to evaluate whether it is higher in the preferred forest habitat and whether there is an effect of parent shrikes on nest predation. Third, we compared the level of shrike nest concealment between habitats and we examined its effect on the nest success of shrikes. Fourth, we conducted a simulated nest predator intrusion experiment to manipulate the perceived risk of nest predation and to measure the level of parental caution during nest visits in both habitat types. We believe that the results provide novel insights into the mechanisms of habitat selection and nest predation in ecologically trapped organisms and we discuss the conservation implications of our findings.

## Materials and Methods

Sampling procedures and experimental manipulations described below were carried out in accordance with the national legislation on the capture (with full release on the spot of capture) of wild birds and approved by the institutional committee on bird protection and nature conservation of the Service Public de Wallonie (DNF/DGARNE). Permission for field experiments with nest predators was granted and approved by the ethical commission of the Royal Belgian Institute of Natural Sciences (https://www.naturalsciences.be).

### Study species and study areas

The Red-backed shrike is an insectivorous migratory bird with a large breeding range across the Western Palearctic [36]. The breeding period is short (May-July) with a single clutch produced; if it fails, shrikes commonly lay a replacement clutch [21,22]. Clutches are frequently depredated by corvids [22]. As raptors and small mammals are only reported anecdotally as nest predators [23,24], we assumed that they were unlikely to play a significant role in our study system.

We worked in two 400-km^2^ study areas in Belgium (centers of study areas: 50°14’N 5°50’E and 49°49’N 5°39’E) that represent mixed farmland-forest landscape. In such a landscape, shrikes breed in two distinct habitat types: (1) farmland covered by pastures and hay meadows with hedgerows and shrubs, and (2) open areas in Norway spruce (*Picea abies*) plantation forests with post-harvesting, early-successional vegetation and young spruce trees (< 10-years old and < 3–4 m height) [15,20]. In Belgium, spruce plantations have been established since 1850 and their cover has increased fourfold over the last century [37].

In total, 58 breeding sites were studied in farmland habitat and 60 breeding sites were studied in forest habitat, both with early-successional and shrubby vegetation as described above. Each breeding site encloses one or a few shrike territories (N = 104 in farmland and N = 109 in forest during the period 2008–2010). Based-on the season-long fieldwork (see below), the boundaries of shrike territories were identified in the field and digitalized in a Geographical Information System. Territory size ranged between 1 and 2 ha both in farmland and in forest. The same territories may be occupied by the same or different shrikes during consecutive years. Previous studies based on data collected in these territories have shown that both nest success and reproductive performance are considerably lower in forest than in farmland [15].

Vegetation composition and structure differ greatly between forest and farmland breeding sites and this difference influences nest site placement in shrikes. In farmland, thorny and dense hedgerows or bushes (e.g. *Rosea sp*., *Prunus sp*. and *Crataegus sp*.) are used as nest sites, whereas young and isolated trees (*Picea abies*) or shrubs (*Sambucus sp*.) are mostly used in forest. As a consequence, available nest sites for shrikes in forest have a more erect and less compact structure compared to farmland.

### Field methods

The overall aim of this study was determining to what extent between-habitat differences in nest predator abundance, nest predation, nest concealment and level of parental caution during nest visits contribute to explaining the observed ecological trap in shrikes.

#### Nest predator abundance during shrike settlement period

In 2009 and 2010, we used a point count survey method to estimate the relative abundance of each corvid species (Magpie *Pica pica*, Eurasian Jay *Garrulus glandarius*, and Carrion Crow *Corvus corone*) in a series of randomly selected shrike territories (N = 43 in farmland and N = 38 in forest) occupied by the shrikes the year before the counts (in 2008 and 2009, respectively). We only used in the subsequent analyses the counts made in territories that were actually occupied by the shrikes for breeding during the same year as the point count survey. Corvid point counts were conducted in April, i.e. shortly before the arrival and settlement of shrikes in early May, thereby reflecting nest predator abundance at the time of habitat selection. We used a corvid survey technique [38] based on the standard point count methods [39]. Each count lasted 10 minutes and all vocal and visual corvid detections were recorded within a 50-m radius of the observer (FAH). Locations of the point count were randomly selected within the boundaries of the shrike territories using a Geographical Information System. Corvid counts were replicated between 3 and 6 times in each territory and each year. The average number of corvid counts in the shrike territories was the same in farmland (N = 3.20 ± 1.69) and in forest (N = 3.28 ± 1.64) (ANOVA: *F*_1,76_ = 0.04, *P* = 0.84). For each year, the relative abundance of each of the three corvid species in each shrike territory was computed as the average number of detections among the different counts conducted in that territory.

#### Seasonal change in nest predator abundance

In 2010, we used the same corvid survey technique as described above to estimate the relative abundance of the corvid species in both habitat types during the breeding period of shrikes in May-July in order to evaluate whether potential between-habitat differences in nest predator abundance change over the course of the season. Between 6 and 15 corvid point counts were conducted during 10 minutes within the same shrike territories as during the settlement period of shrikes. The average number of corvid counts in the shrike territories during the breeding period was the same in farmland (N = 6.77 ± 0.52) and in forest (N = 7.61 ± 0.53) (ANOVA: *F*_1,78_ = 0.05, *P* = 0.83). The relative abundance of each of the three corvid species in each shrike territory was computed in the same way as for point counts conducted during the shrike settlement period.

#### Predation on actual shrike nests

During three consecutive years (2008–2010), we studied nest predation in shrike territories established in farmland (N = 104) and forest (N = 109) [15]. Shrike territories were visited every two days during the whole breeding period (i.e. during the period of first and replacement clutches) to find nest locations and record reproductive activities with binoculars (e.g. nest building, male courtship feeding of the incubating female, nest visits by the parents for food provisioning). Once the nest was located, it was visited every two days to determine the breeding status (i.e. eggs or age of nestlings). Before visiting shrike nests, the presence of corvids in the shrike territories was carefully checked because human nest visitation could increase the probability of nest disclosure for corvids. When nests were found with nestlings, the hatching date was retroactively calculated from the age of nestlings (based on detailed pictures provided in [40]). The disappearance of a complete clutch or the disappearance of nestlings (< 12-days old) between two consecutive nest visits was used as an indicator of nest predation [21,22]. Brood failure due to nest abandonment was recorded when cold eggs or dead nestlings were found in the nest.

#### Predation on artificial nests

We conducted an artificial nest experiment in 2009 and 2010 to obtain baseline estimates of nest predation independent of the behavioural response of parent shrikes to nest predation in both habitat types. We assumed that differences in nest predation between artificial and actual shrike nests were attributable to the parent shrikes [12,41,42]. We estimated these differences in each habitat type separately to evaluate whether shrikes influence nest disclosure for predators more in one habitat type than in the other.

In a number of farmland (N = 26) and forest (N = 25) breeding sites, we placed three man-made nests (tied with dried weeds to mimic shrike nests, see [36]) with two Japanese quail eggs (*Coturnix japonica*) in early June to cover the period of first clutches (clutch sequence = 1) (total number of artificial nests: N = 253). Some of the artificial nests (N = 79) were placed in nest sites used by shrikes for the first clutch in the previous year when this information was available [15] and the others (N = 174) were placed in potentially suitable nest sites according to the expert knowledge of the observer (FAH). Artificial nests were concealed at a similar height (i.e. 2–3 m) and position as actual shrike nests. With this sampling strategy, we were able to mimic shrike nests and to measure nest predation while removing the parental activity [11]. As corvids are known to have good memory skills with regard to the position of depredated nest sites [43] and as shrikes use different nest sites between first and replacement clutches [44], artificial nests were removed in mid of June and replaced with new ones (N = 242) in other nest sites within the same breeding sites to cover the period of replacement clutches (clutch sequence = 2) until late June. Nest sites were the same as those used by shrikes for their replacement clutches in the previous year if they laid a replacement clutch (N = 34) or potentially suitable nest sites (N = 208). Nest building and egg placement was done by one and the same observer (FAH) with latex gloves to avoid human odor traces [45]. Artificial nests were visited every two days and nest predation was considered when quail eggs disappeared or were broken. Again, the presence of corvids in the breeding sites was carefully checked before placing or visiting the artificial nests.

In 2010, an additional set of artificial nests (N = 158 in farmland and N = 155 in forest) were baited with four plasticine eggs to verify whether nest predators were indeed mainly corvids in our study system or alternatively also mammals [22].

#### Nest concealment

In 2008–2010, actual shrike nest concealment was assessed in farmland (number of nests examined: N = 127) and in forest (N = 134) during first (clutch sequence = 1) and replacement (clutch sequence = 2) clutches. Each nest was screened visually at 1-m distance in the four cardinal directions and from a similar height to the nest itself [46]. The proportion of the nest estimated as visible was averaged across the four cardinal directions. Based on this information, each nest was assigned to one of three categories according to a multinomial concealment score attributed by one and the same observer (FAH): 1 = low concealment (≥ 2/3 of the nest is visible), 2 = moderate concealment (1/3 to 2/3 of the nest is visible), and 3 = high concealment (≤ 1/3 of the nest is visible). For successful nests, concealment was evaluated at the time of fledging (nestlings between 12 and 15 days old); for unsuccessful nests, this was done within two days after brood failure.

#### Level of caution during parental nest visits under simulated nest predator intrusion

Through nest visits for food provisioning, parent birds may disclose nest location to nest predators [11]. Parent birds may therefore modify the nest visitation rate according to the perceived predation risk when visiting their nests [13]. In addition, this level of caution during nest visits is known to depend on the overall vegetation type in the vicinity of the nest site [11,13]. As breeding sites in farmland and forest habitat types strongly differ with regard to their vegetation structure, this may lead to between-habitat differences in nest concealment and parent shrikes may adjust their level of caution to counterbalance this effect. Hence, there is a reason to evaluate the level of parental caution in both habitat types.

To do so, we measured nest visitation rate by parent shrikes before and during a simulated nest predator intrusion using a video-recording technique [13]. The experiment was conducted between 8–9 am and 11–12 am during the second part of June 2010 and on shrike nests hosting 12-day old nestlings (range 11–15 days) in a number of randomly selected shrike territories within farmland (N = 26) and forest (N = 29) sites. A stuffed nest predator (Jay, Magpie or Crow) was randomly selected and perched at *c*. 2-m above the ground on a stick at a 15-m distance from the nest. To avoid a confounded effect of the activities of the experimenter (FAH) and the simulated nest predator intrusion, we placed the stuffed predator at the same time as the video-recording equipment but we covered it with a black plastic box [13]. We started the video recording of the nest in the absence of a nest predator 2 hours after the equipment was placed and for a 30-minute period. Next, we removed the plastic box with a remote control and we video-recorded the nest in the simulated presence of a nest predator during 30 minutes. This simulated nest predator intrusion experiment was carried out with Jay (N = 20), Magpie (N = 17) and Crow (N = 18) as stuffed nest predator. The change in nest visitation rate was calculated as the ratio between the mean duration for the parents to visit their nest ten times after the first visit during and before nest predator exposure. An increase in the level of parental caution during predator exposure was assumed to decrease the nest visitation rate relative to the situation without nest predator intrusion.

### Statistical analyses

Table 1 synthesizes the models used in the analyses to test the between-habitat differences in nest predator abundance, nest predation, nest concealment and level of parental caution during nest visits. First, we tested whether nest predator abundance differed between forest and farmland (Table 1, section A) and whether potential differences changed between shrike settlement and breeding periods (Table 1, section B). Second, we tested for between-habitat differences in nest predation on actual shrike nests (with parental effect, Table 1, section C) and on artificial nests (without parental effect, Table 1, section D). Third, we compared the level of nest concealment between habitat types (Table 1, section E) and we examined the link between nest concealment and nest predation (Table 1, section F). Fourth, we tested whether the level of caution of parent shrikes when visiting their nests during the simulated nest predator intrusion differed between habitat types (Table 1, section G).

**Table 1:** Fixed and random effects used in the GLMM (sections A-F) and GLM (section G) analyses of nest predator abundance, nest predation, nest concealment and the level of parental caution during nest visits.

Section	N	Response	Fixed effects													Random effects	Distribution
			Habitat	Clutch sequence	Year	Study area	Nest concealment	Nest predator	Season	Habitat x Clutch sequence	Habitat x Year	Habitat x Study area	Habitat x Nest concealment	Habitat x Nest predator	Habitat x Season		
A	253	Nest predator abundance^a^ ^,^ ^b^	X		X	X					X	X				Territory	Poisson
B	727	Nest predator abundance^a^ ^,^ ^c^	X			X			X			X			X	Territory	Poisson
C	438	Predation on actual shrike nests	X		X	X					X	X				Site	Binomial
D	495	Predation on artificial nests	X		X	X					X	X				Site	Binomial
E	261	Nest concealment	X	X	X	X				X	X	X				Site	Multinomial
F	261	Predation on actual shrike nests^d^	X				X						X			Site	Binomial
G	55	Level of parental caution	X				X	X					X	X		-	Normal

N (sample size) = number of corvid point counts (sections A-B) or number of nests examined (sections C-G).

Covariates: recording date (sections A-B), clutch sequence (sections C-D), nestling age and brood size (section G).

^a^Analysis carried out with all nest predators together (see Tables 2 and 3) and with each nest predator species separately (see Results section).

^b^Analysis carried out with corvid point counts conducted during the shrike settlement period in 2009 and 2010.

^c^Analysis carried out with corvid point counts conducted during the shrike settlement and breeding periods in 2010 to examine seasonal change in nest predator abundance.

^d^As nest concealment was estimated in only part of shrike nests, we used a simpler model structure than in section C to explore how nest concealment influences nest predation in both habitat types.

We used generalized linear models (GLMs with restricted Maximum Likelihood estimations) when a single observation in each breeding site and in each shrike territory was taken into account (Table 1, section G) and generalized linear mixed models (GLMMs with Maximum Likelihood estimations) with site or territory as random effect when several observations in each breeding site or in each shrike territory were included in the analyses (Table 1, sections A-F). In addition to the factor ‘Habitat’ (i.e. farmland or forest) included as a fixed effect in the models, we also accounted for the variation of the response variables over time (between-year variation ‘Year’ and within-year variation ‘Clutch sequence’ and ‘Season’) and in space (Study area). Interaction terms (‘Habitat x Year’, ‘Habitat x Clutch sequence’, ‘Habitat x Season’, ‘Habitat x Study area’) were also included to test whether the between-habitat differences in the response variables were consistent over time and in space. ‘Nest predator’ (i.e. Jay, Magpie or Crow) and its interaction with ‘Habitat’ were included in the analysis of the level of parental caution during nest visits to test for between-predator differences in parental caution [47] and for consistency between habitat types. Recording date, clutch sequence, nestling age and brood size were included as covariates to control for their effect on the response variables when needed. All explanatory variables were standardized (mean = 0 and SD = 1) and analyses were performed using the R 2.13. [48] and SAS 9.2. [49] software.

Information-theoretic multimodel inference was used based on the Akaike’s Information Criterion corrected for small sample sizes (AIC_c_). ΔAIC_c_ were calculated for each candidate model to reflect the difference with the best candidate model (i.e., the model with the smallest AIC_c_). A ΔAIC_c_ value < 2 was used as a threshold for a model to receive some support [50]. Relative support for alternative models was obtained by scaling them according to AIC_c_ weight [50]. The relative importance of a variable (*w*_*+*_) was estimated by summing the AIC_c_ weights across all candidate models in which the variable occurred. The number of times a variable is included in the different candidate models varied from one variable to the other: some of them are included only in a few models, such as interaction terms that are included only when both parent variables are also included. This prevalence of the variables in the candidate models (ν) restricted the *w*_*+*_ values estimated for the different variables within the multimodel inference framework [50]: variables with low ν values are more likely to obtain low *w*_*+*_ values than variables with high ν values. For this reason, ν values are reported as a baseline reference along with the *w*_*+*_ values in the results. The model-averaged parameter estimates (β), the precision of the estimates (unconditional standard errors, S.E.) and the *w*_*+*_ values are reported to indicate the importance of each variable for explaining variation in the response variable [50]. Parameter estimates were also converted into percentages relative to the average value of the response variables (Δ[difference between two levels of a variable]).

## Results

The sets of supported models for each analysis (sections A-G in Table 1) are included in Table 2.

**Table 2:** Set of supported and best non-supported candidate models along with their respective support according to the model selection procedures.

Section	Response	Supported and (best non-supported) models	K	Log Likelihood	ΔAIC_c_	AIC_c_ weight
**A**	**Nest predator abundance (during shrike settlement period)**	Habitat + Year + Study area + Habitat x Study area	7	-209.73	0.00	0.61
		Habitat + Year + Study area + Habitat x Year + Habitat x Study area	8	-209.57	1.82	0.25
		(Habitat + Year)	(5)	(-213.97)	(4.28)	(0.07)
**B**	**Nest predator abundance (during shrike settlement and breeding periods)**	Habitat + Study area + Habitat x Study area	6	-443.03	0.00	0.43
		Habitat + Study area + Season + Habitat x Study area + Habitat x Season	8	-441.035	0.10	0.41
		(Habitat + Study area + Season + Habitat x Study area)	(7)	(-443.03)	(2.04)	(0.16)
**C**	**Predation on actual shrike nests**	Habitat + Year + Study area+ Habitat x Study area	8	-246.81	0.00	0.53
		Habitat + Year + Study area	7	-248.57	1.45	0.26
		(Habitat + Year + Study area + Habitat x Year + Habitat x Study area)	(10)	(-246.78)	(4.11)	(0.07)
**D**	**Predation on artificial nests**	Habitat + Year + Study area	5	-274.24	0.00	0.23
		Habitat + Year + Study area + Habitat x Year	6	-273.40	0.36	0.19
		Year + Study area	4	-275.54	0.56	0.18
		Habitat + Year + Study area + Habitat x Study area	6	-274.08	1.73	0.10
		(Habitat + Year)	(4)	(-276.29)	(2.05)	(0.08)
**E**	**Nest concealment**	Habitat + Clutch sequence + Habitat x Clutch sequence	5	-254.60	0.00	0.24
		Habitat + Clutch sequence	4	-255.66	0.05	0.24
		Habitat + Study area + Clutch sequence	5	-255.59	1.98	0.09
		(Habitat + Study area + Clutch sequence + Habitat x Clutch sequence)	(6)	(-254.56)	(2.03)	(0.09)
**F**	**Predation on actual shrike nests**	Nest concealment	4	-138.34	0.00	0.44
		Habitat + Nest concealment	5	-137.48	0.36	0.37
		(Intercept)	(2)	(-141.91)	(3.03)	(0.10)
**G**	**Level of parental caution**	Nest concealment	6	-32.67	0.00	0.51
		Habitat + Nest concealment	7	-29.26	1.54	0.23
		Intercept	4	-29.02	1.35	0.18
		(Habitat)	(5)	(-31.01)	(3.79)	(0.08)

The correspondence with the different sections of Table 1 is indicated with a capital letter (sections A-F). ΔAIC_c_ refers to the differences in AIC_c_ between the model and the best candidate model associated with the smallest AIC_c_. All supported models (ΔAIC_c_ < 2) are reported and the best non-supported models (ΔAIC_c_ > 2) are indicated between brackets. AIC_c_ weight indicates the relative support for each model within each section. The number of parameters (K) is reported for each model.

### Nest predator abundance during shrike settlement period

**Fig 1:**
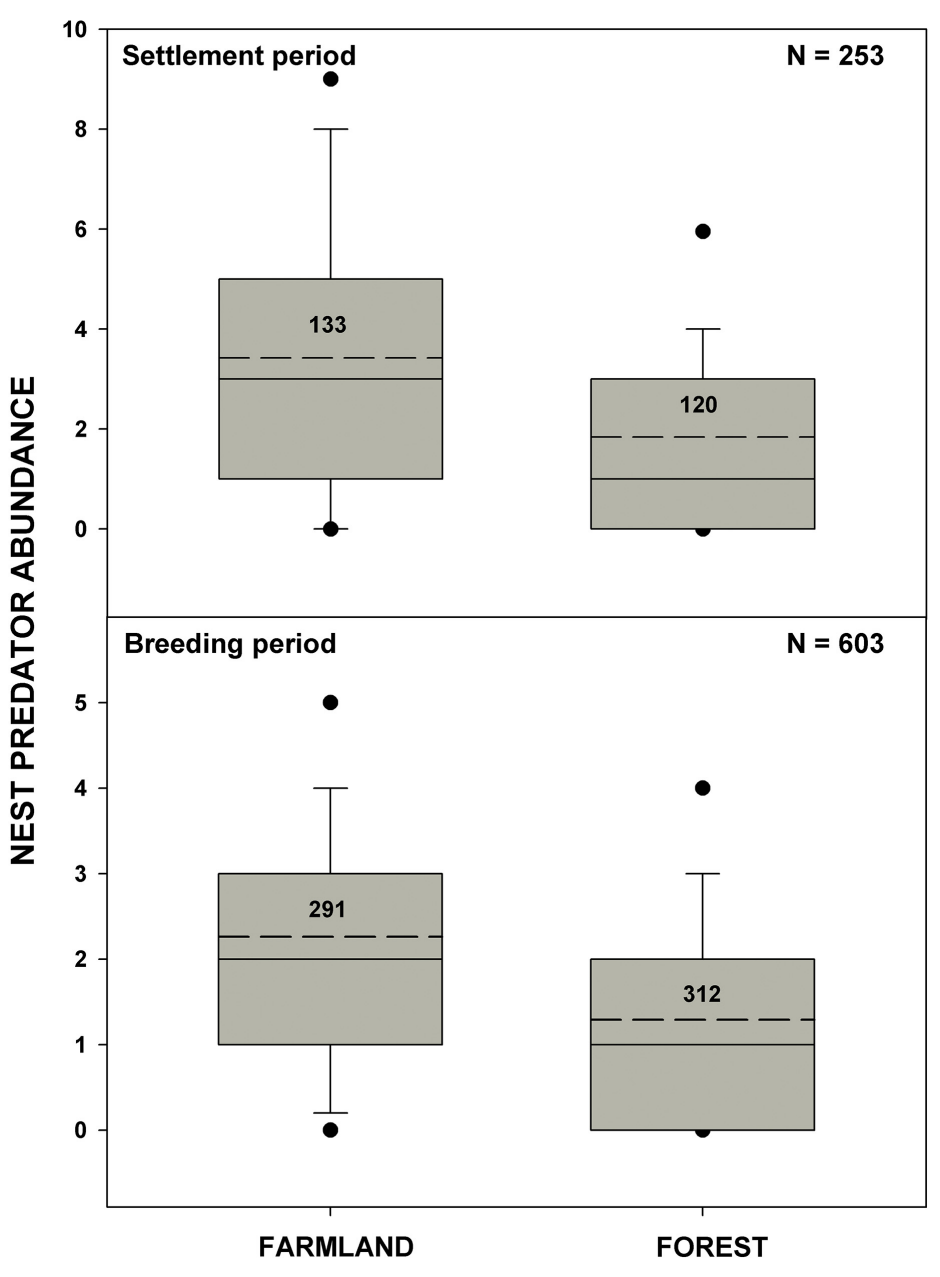
Nest predator abundance in farmland and forest. Nest predator abundance is the number of Jays, Magpies and Crows detected during the point count survey in shrike territories within farmland and forest habitats during the period of shrike settlement and breeding. Box-and-whisker plots represent 5^th^ and 95^th^ percentiles [┴ and ┬], min-max [•], mean [––] and median [−]) values. Sample sizes refer to the number of corvid point counts.

During the shrike settlement period, we counted on average almost twice as many corvids in shrike territories established in farmland as in forest (Fig 1, Table 3, section A, ‘Habitat’: *w*_*+*_ = 100%, Δ[Farmland-Forest] = 53%). Nest predator abundance also differed between years (*w*_*+*_ = 100%, Δ[2009–2010] = 49%) and study areas (*w*_*+*_ = 90%, Δ[Study area 1-Study area 2] = 13%). Although nest predator abundance was much higher in farmland than in forest in each study area, the between-habitat difference was larger in study area 1 than in study area 2 (interaction effect ‘Habitat x Study area’: *w*_*+*_ = 86%). The between-habitat difference in nest predator abundance did not change between years (interaction effect ‘Habitat x Year’: *w*_*+*_ = 29%).

**Table 3:** Results of the AIC_c_-weighted GLMM (sections A-F) and GLM (section G) model selection procedures for the analyses of nest predator abundance (all corvid species together; A, B), nest predation (C, D and F), nest concealment (E) and the level of parental caution during nest visits (G).

Section	Response						
**A**	**Nest predator abundance (during shrike settlement period)**						
	**Fixed effect**		ν	***w***_***+***_	**β**	**S.E.**	**Effect**
	*(Intercept)*		*100*	*100*	*1*.*56*	*0*.*17*	
	**Habitat**	(Forest)	69	100	-1.10	0.28	Farmland > Forest
	**Year**	(2010)	62	100	-0.67	0.10	2009 > 2010
	**Study area**	(2)	62	90	-0.23	0.17	1 > 2
	**Habitat x Year**	(Forest, 2010)	23	29	-0.03	0.06	
	**Habitat x Study area**	(Forest, 2)	23	86	0.71	0.26	Farmland 1 > Farmland 2 > Forest 2 > Forest 1
**B**	**Nest predator abundance (during shrike settlement and breeding periods)**							
	**Fixed effect**		ν	***w***_***+***_	**β**	**S.E.**	**Effect**
	*(Intercept)*		*100*	*100*	*0*.*92*	*0*.*08*	
	**Habitat**	(Forest)	60	100	-0.97	0.14	Farmland > Forest
	**Study area**	(2)	60	100	-0.31	0.12	1 > 2
	**Season**	(Breeding period)	62	57	-0.09	0.11	
	**Habitat x Study area**	(Forest, 2)	20	100	0.74	0.18	Forest 1 > Farmland 1 > Forest 2 > Farmland 2
	**Habitat x Season**	(Forest, Breeding period)	23	41	0.14	0.11	
**C**	**Predation on actual shrike nests**						
	**Fixed effect**		ν	***w***_***+***_	**β**	**S.E.**	**Effect**
	*(Intercept)*		*100*	*100*	*0*.*28*	*0*.*36*	
	**Habitat**	(Forest)	70	96	0.87	0.38	Forest > Farmland
	**Year**	(2009)	62	95	-0.93	0.32	2008 > 2010 > 2009
		(2010)	62	95	-0.66	0.30	
	**Study area**	(2)	62	95	-0.80	0.35	1 > 2
	**Habitat x Year**	(Forest, 2009)	23	10	-0.01	0.07	
		(Forest, 2010)	23	10	-0.02	0.06	
	**Habitat x Study area**	(Forest, 2)	23	62	0.51	0.34	Forest 1 > Farmland 1 > Forest 2 > Farmland 2
**D**	**Predation on artificial nests**						
	**Fixed effect**		ν	***w***_***+***_	**β**	**S.E.**	**Effect**
	*(Intercept)*		*100*	*100*	*-0*.*71*	*0*.*38*	
	**Habitat**	(Forest)	77	76	-0.34	0.36	Farmland > Forest
	**Year**	(2010)	63	100	2.14	0.34	2010 > 2009
	**Study area**	(2)	63	77	0.52	0.33	2 > 1
	**Habitat x Year**	(Forest, 2010)	26	35	-0.23	0.23	
	**Habitat x Study area**	(Forest, 2)	26	18	0.07	0.14	
**E**	**Nest concealment**						
	**Fixed effect**		ν	***w***_***+***_	**β**	**S.E.**	**Effect**
	*(Intercept)*	*(Nest concealment = 2)*	*100*	*100*	*0*.*74*	*0*.*27*	
		*(Nest concealment = 3)*	*100*	*100*	*-0*.*92*	*0*.*27*	
	**Habitat**	(Forest)	77	100	-0.79	0.31	Farmland > Forest
	**Year**	(2009)	63	45	-0.08	0.11	
		(2010)	63	45	-0.01	0.08	
	**Study area**	(2)	63	33	-0.02	0.09	
	**Clutch sequence**	(2)	63	98	-0.44	0.35	1 > 2
	**Habitat x Year**	(Forest, 2009)	26	6	-0.002	0.18	
		(Forest, 2010)	26	6	-0.005	0.02	
	**Habitat x Study area**	(Forest, 2)	26	9	-0.01	0.05	
	**Habitat x Clutch sequence**	(Forest, 2)	26	89	-0.35	0.31	Farmland 1 > Farmland 2 > Forest 1 > Forest 2
**F**	**Predation on actual shrike nests**						
	**Fixed effect**		ν	***w***_***+***_	**β**	**S.E.**	**Effect**
	*(Intercept)*		*100*	*100*	*0*.*71*	*0*.*29*	
	**Habitat**	(Forest)	60	46	0.18	0.18	
	**Nest concealment**	(2)	60	86	-0.71	0.33	1 > (2 = 3)
		(3)	60	86	-0.71	0.40	
	**Habitat x Nest concealment**	(Forest, 2)	20	5	-0.01	0.04	
		(Forest, 3)	20	5	0.01	0.05	
**G**	**Level of parental caution**						
	**Fixed effect**		ν	***w***_***+***_	**β**	**S.E.**	**Effect**
	*(Intercept)*		*100*	*100*	*0*.*30*	*0*.*11*	
	**Habitat**	(Forest)	77	33	-0.04	0.03	
	**Nest predator**	(Jay)	63	18	-0.01	0.05	
		(Magpie)	63	18	-0.02	0.04	
	**Nest concealment**	(2)	63	79	-0.04	0.03	1 > 2 > 3
		(3)	63	79	-0.38	0.10	
	**Habitat x Nest predator**	(Forest, Jay)	26	4	0.007	0.02	
		(Forest, Magpie)	26	4	0.011	0.03	
	**Habitat x Nest concealment**	(Forest, 2)	26	2	0.003	0.01	
		(Forest, 3)	26	2	0.007	0.04	

The AIC_c_-weighted relative importance (*w*_*+*_), the model-averaged parameter estimates (β) and the unconditional standard error (S.E.) are reported for each variable (main effects and interactions), as well as their prevalence in the set of candidate models (ν). The parameter estimates refer to the level indicated between brackets as a baseline. The interpretation of each effect is provided in case of AIC_c_-based support only. The correspondence with Table 1 is indicated with a capital letter (sections A-G).

Separate analyses for each corvid species showed that higher abundance in farmland was especially due to the Carrion Crow (mean number of individuals per count session: 1.74, ‘Habitat’: β = -1.58 ± 0.39, *w*_*+*_ = 100%, Δ[Farmland-Forest] = 79%) and, to a lesser extent, to the Magpie (mean number of individuals per count session: 0.20, ‘Habitat’: β = -0.64 ± 0.13, *w*_*+*_ = 100%, Δ[Farmland-Forest] = 26%). There was low support for slightly higher abundance of Jay in the forest habitat (mean number of individuals per count session: 0.70, ‘Habitat’: β = 0.24 ± 0.21, *w*_*+*_ = 72%, Δ[Farmland-Forest] = -12%).

### Seasonal change in nest predator abundance

Although the overall number of corvids recorded during the shrike breeding period was slightly lower than that during the settlement period (Fig 1, Table 3, section B, ‘Season’: *w*_*+*_ = 57%, Δ[Settlement period-Breeding period] = 16%), there was relatively low support for seasonal change in the between-habitat differences in nest predator abundance (interaction effect ‘Habitat x Season’: *w*_*+*_ = 41%). Hence, the number of corvids remained higher in farmland than in forest (‘Habitat’: *w*_*+*_ = 100%, Δ[Farmland-Forest] = 81%) over the course of the season (Fig 1).

The overall higher nest predator abundance in farmland during the breeding period of shrikes was observed in all corvid species: the Carrion Crow (mean number of individuals per count session: 1.09, Habitat’: β = -1.14 ± 0.27, *w*_*+*_ = 100%, Δ[Farmland-Forest] = 89%), the Magpie (mean number of individuals per count session: 0.15, Habitat’: β = -0.22 ± 0.06, *w*_*+*_ = 98%, Δ[Farmland-Forest] = 22%), and the Jay (mean number of individuals per count session: 0.52, Habitat’: β = -0.36 ± 0.22, *w*_*+*_ = 91%, Δ[Farmland-Forest] = 31%). For each corvid species, the higher abundance in farmland was observed during both the settlement period and the breeding period (interaction effect ‘Habitat x Season’: *w*_*+*_ < 10% in all models for the different corvid species).

### Predation on actual shrike nests

In 2008–2010, we recorded 183 brood failures among the 438 breeding attempts that we surveyed. Among the brood failures, 170 (93%) resulted from nest predation, 11 nests (6%) were abandoned during incubation and 2 nests (1%) were found with dead nestlings. Hence, the overall nest predation rate (i.e. the proportion of predation-attributed brood failures among the breeding attempts) was estimated at *c*. 39%.

Nest predation on shrike nests was higher in forest (nest predation rate estimated at *c*. 42%) than in farmland (*c*. 35%) (Table 3, section C, ‘Habitat’: *w*_*+*_ = 96%, Δ[Farmland-Forest] = -25%). There was some support for a year effect (‘Year’: *w*_*+*_ = 95%, Δ[2008–2009] = 36%, Δ[2008–2010] = 33%) and an effect of study area (‘Study area’: *w*_*+*_ = 95%, Δ[Study area 1-Study area 2] = 33%). There was also support for an interaction effect (‘Habitat x Study area’: *w*_*+*_ = 62%) because the between-habitat difference in nest predation was stronger in study area 2 than in study area 1.

### Predation on artificial nests

Among the artificial nests with plasticine eggs, 48% had at least one egg with beak marks and the remaining ones had no mark; none of them were found with mammal teeth marks. In contrast with actual shrike nests, predation on artificial nests with quail eggs was higher in farmland (nest predation rate estimated at *c*. 68%) than in forest (*c*. 55%) (Table 3, section D, ‘Habitat’: w_+_ = 76%, Δ[Farmland-Forest] = 13%). Artificial nest predation varied substantially between years (‘Year’: w_+_ = 100%, Δ[2009–2010] = -38%) and study areas (‘Study area’: w_+_ = 77%, Δ[Study area 1-Study area 2] = -12%), but the between-habitat difference in artificial nest predation was similar between years and study areas (interaction effects ‘Habitat x Year’ and ‘Habitat x Study area’: all w_+_ < 35%).

### Nest concealment

**Fig 2:**
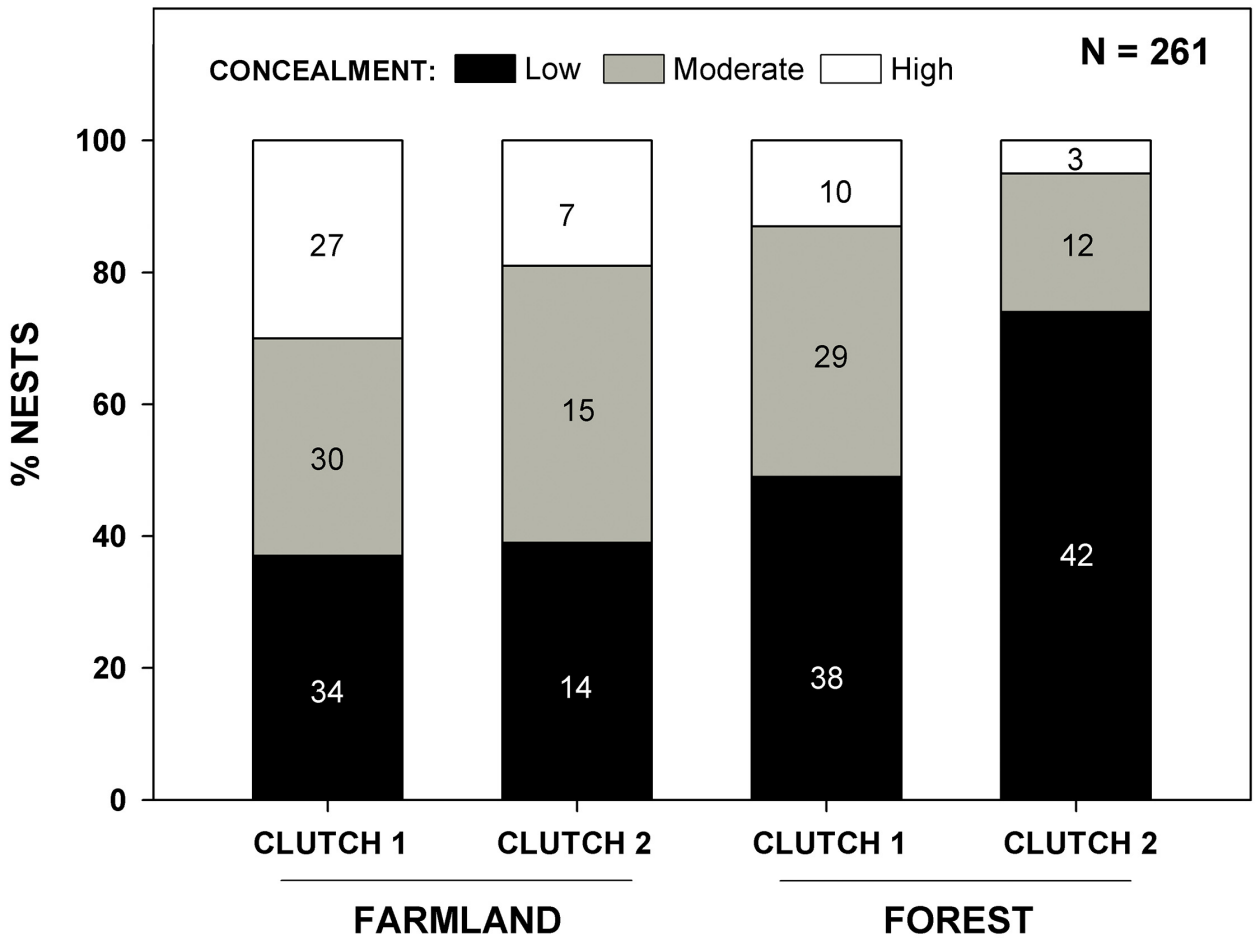
Nest concealment in farmland and forest. Percentage of nests in farmland and forest habitats and in first (clutch 1) and replacement (clutch 2) clutches assigned to one of three categories of nest concealment: low, moderate and high concealment. Sample sizes refer to the number of nests used to measure nest concealment.

Nests were more frequently poorly concealed (low to moderate concealment: scores 1 and 2) in forest than in farmland (Table 3, section E, ‘Habitat’: *w*_*+*_ = 100%) and in replacement than in first clutches (‘Clutch sequence’: *w*_*+*_ = 98%). Low nest concealment was particularly prevalent in replacement clutches and in forest sites (Fig 2), explaining the important interaction between habitat and clutch sequence (interaction effect ‘Habitat x Clutch sequence’: *w*_*+*_ = 89%). There was no difference in nest concealment between years (‘Year’: *w*_*+*_ = 45%) or between study areas (‘Study area’: *w*_*+*_ = 33%). The between-habitat difference in nest concealment was consistent between years and study areas (interaction effects ‘Habitat x Study area’ and ‘Habitat x Year’: all *w*_*+*_ < 10%).

Predation on actual shrike nests decreased with increasing levels of nest concealment (Table 3, section F, ‘Nest concealment’: *w*_*+*_ = 86%): nests with high (score 3) and moderate (score 2) concealment were less frequently depredated than nests with low concealment (score 1). This result was the same in both habitat types (interaction effect ‘Habitat x Nest concealment’: *w*_*+*_ = 5%).

### Level of caution during parental nest visits under simulated nest predator intrusion

In the presence of a simulated nest predator, the nest visitation rate decreased threefold compared to the visitation rate in the absence of a nest predator (mean level of parental caution = 3.1 ± 0.6). This increase in the level of parental caution was similar in both habitat types (Table 3, section G, ‘Habitat’: *w*_*+*_ = 33%) and for the different nest predator species (‘Nest predator’: *w*_*+*_ = 18%). In addition, there was no interaction between habitat types and nest predator species (interaction effect ‘Habitat x Nest predator’: *w*_*+*_ = 4%). There was considerable support for a lower level of parental caution in well-concealed nests (‘Nest concealment’: *w*_*+*_ = 79%, Δ[high nest concealment-low nest concealment] = 76%) and this difference was the same in both habitat types (interaction effect ‘Habitat x Nest concealment’: *w*_*+*_ = 2%).

## Discussion

Over recent decades, changes in forest harvesting techniques have created rotational systems of large-sized, open areas in plantation forests attracting early-successional wildlife [51]. Several so-called ‘farmland birds’ have recently extended their habitat use into these post-harvesting, early-successional areas in spruce plantation forests [52,53]. However, this novel habitat type constitutes an ecological trap for the Red-backed shrike: this passerine bird species prefers breeding in the open areas in forest, even if nest success is considerably lower than in the traditionally used farmland sites [15].

Here, we showed that lower nest success in the novel, preferred forest habitat results from higher nest predation from corvids even if these nest predators are much more abundant in farmland. As corvid abundance was higher in farmland during both the period of shrike territory settlement and the breeding period of the shrikes, this ruled out the ‘numerical response’ hypothesis of a seasonal change in nest predator abundance. Instead, our results are consistent with the alternative ‘functional response’ hypothesis of a decoupling between nest predator abundance and nest predation. In contrast with most reported cases of ecological traps showing that organisms fail to avoid areas with high nest predator abundance [10] and have lower nest success in the preferred habitat type hosting more nest predators [9,54], we argue that the functional response of nest predators rather than their numerical response may underlie lower nest success in a preferred habitat type.

Nest predation may deviate from nest predator abundance due to complex interactions between habitat-specific vegetation structure, food resources and foraging behaviour of both the nest predators and the prey [11,31,32,55–57]. At this stage, we are not yet able to identify the full range of mechanisms that underlie higher nest predation in a habitat with fewer nest predators. The interplay between food limitations for the shrikes [19,20] and nest predation in this study system clearly warrants further investigation and experimental work [58] because both could act in tandem to attract shrikes to the forest habitat and reduce their nest success. It has been argued that nest predators generally find nests by chance [59], but they may also learn locating the nests and, hence, use cues that guide them [60]. Nest visits of the parent birds to deliver food to their offspring may, for instance, direct predators to nests [11]. Our results on artificial nest predation (without parental effect) indicate than corvids search for nest sites more intensively in farmland than in forest. The opposite pattern of predation on actual shrike nests (with parental effect) therefore indicates that parent shrikes influence nest predation and facilitate nest disclosure for predators in forest more than they do in farmland [12]. Based on the results from the simulated nest predator experiment, we could not show that the level of parental caution in the presence of a predation risk was different in forest and in farmland. However, habitat structure in forest breeding sites may increase exposure to nest predation because it could be easier for visually-oriented nest predators to track parents during nest visits as a cue to the location of the nest [31]. Further field experiments are needed to test such hypothesis.

It is now warranted to identify which nest predator species is the most relevant for shrikes. Although our results have confirmed the significant role of corvids in the study system, we could not evaluate separately the effect of the different corvid species. Even if the level of parental caution was similar in the presence of each of the three potential nest predators, the different corvid species differ in their foraging strategies [61], which could influence the likelihood to find shrike nests. For example, Jays are cryptic sit-and-wait predators and may forage at the edge of forests looking for nest activity, whereas Magpies often forage on the ground in open areas and might therefore detect shrike nests in a different way.

The likelihood of nest disclosure for predators may also be higher in forest because shrike nests are poorly concealed compared to farmland. Our results clearly show that between-habitat differences in nest concealment provide, at least partly, a functional explanation for the mismatch between nest predator abundance and nest predation and, hence, for the existence of an ecological trap. We acknowledge, however, that the mechanisms explaining between-habitat differences in nest concealment remain to be identified. For instance, the investment of parent birds in anti-predation strategies has been shown to relate to perceived nest predation pressure [62,63]; this may contribute to explaining why nests are better concealed in the farmland habitat with higher nest predator abundance during shrike settlement and breeding. Alternatively, the opportunities for nest building may greatly differ between habitats due to the structural properties of the vegetation: available vegetation for nest building in forest has a less compact structure and this may explain the lower nest concealment than in farmland.

In line with Roos and Pärt [24], we assumed that the use of nest predator abundance operates as a proximate indicator of habitat quality during habitat selection in the Red-backed shrike. This remains to be tested with choice experiments [64] in our study system where there is an ecological trap at work. Currently, we are not able to rule out the possibility that the forest habitat attracts shrikes based on a lower perceived predation risk for adults. Adult survival is another life history trait that may differ between habitats. It may have a strong impact on habitat selection and population growth in migratory birds [65]. An estimation of habitat-specific adult and first-year survival rates based on long-term data and predation experiments during the breeding period in our study system would help understand habitat preference relative to a more complete assessment of fitness including measures of local recruitment, lifetime reproductive success and population growth rates [15].

The management practices of creating large-sized open areas in plantation forests have been shown to increase nest predation in open-cup nesting species compared to open areas in natural forests [66,67]. However, to the best of our knowledge, only Rodewald [68] compared the effect of forestry and farmland management on nest success in birds. She observed that nest success of several songbirds was lower in forest than in farmland due to higher nest predation. In the same vein, our results indicate that post-harvesting, early-successional areas in spruce plantation forests may provide less valuable breeding conditions for farmland birds than previously suggested based on bird presence data only [52]. In light of our results, it is important to note that this pattern is not due to higher densities of nest predators in the forest breeding sites but to the fact that predators are more effective in locating nests of their prey. Hence, our results draw attention to the potential of forestry practices to create ecological traps for bird species from the farmland community through a mismatch between the abundance of nest predators and their impacts.
